# ERGA-BGE reference genome of *Gambusia holbrooki*, a globally invasive freshwater fish

**DOI:** 10.12688/openreseurope.22693.1

**Published:** 2026-02-13

**Authors:** Bonnie Fraser, Clelia Gasparini, Francesco Santi, Astrid Böhne, Rita Monteiro, Thomas Marcussen, Rebekah A. Oomen, Torsten H. Struck, Wellcome Sanger Institute Tree of Life Management, Samples and Laboratory Team, Aarushi Vaidya, Abitha Thomas, Adam Bates, Aleksandra Bliznina, Alex Makunin, Amit Vishwakumar, Amy Denton, Andy Griffiths, Anna Kovalevskaia, Arif Maulana, Benjamin Jackson, Cam Muyo, Caroline Howard, Charlotte Wright, Chloe Leech, Chris Laumer, Clare Cornwell, Claudia Weber, David Rowland, Ed Symons, Edel Sheerin, Elizabeth Sinclair, Ellen Cameron, Emma Teeling, Emmelien Vancaester, Erna King, Filipa Sampaio, Gene Myers, Graeme Oatley, Haddijatou Mbye, Halyna Yatsenko, Haoyu Niu, Ian Still, Isabelle Clayton-Lucey, Jack Monaghan, Jamie Davis, Jess Bernard, Jessica Thomas-Thorpe, Jessie Jay, Joana Meier, Jonah Walker, Juan Pablo Narváez Gomez, Kamil S Jaron, Keith Porter, Kerstin Howe, Lauma Ramona, Leah Bacon, Lewis Stevens, Liam Prestwood, Lora Downes, Lucy Kitchin, Luke Lythgoe, Maja Todorovic, Manuel Batista, Manuela Kieninger, Mara Lawniczak, Marcela Uliano-Silva, Maria Morra, Mark Blaxter, Martha Mulongo, Matthew Berriman, Max Brown, Molly Carter, Nancy Holroyd, Nicola Chapman, Paul Flicek, Priyanka Sethuk Raman, Radka Platte, Raquel Juliana Vionette do Amaral, Rebecca O'Brien, Richard Durbin, Robina Heathcote, Sam Ebdon, Sinead Calnan, Sophie Potter, Stephanie Fagan, Theodora Anderson, Victoria McKenna, Witek Morek, Yan Liang, Wellcome Sanger Institute Scientific Operations: Sequencing Operations, Abby Crackett, Abdulrahman Tuameh, Alexander Dove, Alexander Hatton, Alice Linsdell, Ana Monteiro, Barbora Pardubska, Ben Farr, Callum Murray, Carlos Jimenez Verdejo, Caroline Mitchell, Chris Henderson, Craig Corton, Danni Weldon, Elizabeth Easthope, Elliott Trigg, Emily Abraham, Emily Gallagher, Emma Dawson, Emma Memune Taluy, Esther Mellado Gomez, Filipa Sampaio, Francesco Iacoviello, Hannah Hanks, Harriet Johnson, Harriet Ninsiima, Henry Mallalieu, Hermione Blomfield-Smith, Ifeoluwapo Joshua, Iraad Bronner, Irene Fabiola Roman Maldonado, Jacqui Brown, James Du Preez, James Mack, James Uphill, James Watts, John Tushabe, Karen Oliver, Karolina Kujawa, Leanne Morrow, Lesley Shirley, Lucy Kitchin, Maariyah Rashid, Mary-Ann Santosh, Mia Franulovic, Michael A. Quail, Michelle Smith, Naomi R. Park, Neil Marriot, Nicholas Redshaw, Paul Heath, Ritoza Das, Robert Newell, Robin Moll, Sally Linsdell, Sarah Holmes, Scott Thurston, Shelly-Ann Coutts, Sophia Uvarova, Tavis Mason, Timi Adewumi, Tobi Ajenifuja, Tracey-jane Chillingworth, Tristram Bellerby, William Knight, Yousra Belattar, Zoe Goate, Wellcome Sanger Institute Tree of Life Core Informatics Team, Shane A McCarthy, Eerik Aunin, Jim Downie, William Eagles, Noah Gettle, James Gilbert, Ksenia Krasheninnikova, Damon-Lee Pointon, Nathan Riley, Ying Sims, James Torrance, Marcela Uliano-Silva, Chenxi Zhou, Jonathan Wood, Dominic Absolon, Karen Brooks, Joanna Collins, Karen Houliston, Michael Paulini, Sarah Pelan, Thomas Mathers, Camilla Santos, Danil Zilov, Matthieu Muffato, Beth Yates, Tyler Chafin, Cibele Gomes de Sotero Caio, Cibin Sadasivan Baby, Ene Goktan, Paul Davis, Priyanka Surana, Zaynab Butt, Ashish Mittal, Charlie Hathaway, Edward Moulsdale, Kiernan Harding, Logan Howat, Luke Wilson, Ahmad Zuheir bin Zaidon, Amy Denton, Caroline Howard, Kerstin Howe, Mark Blaxter, Shane McCarthy, Jonathan M.D. Wood, Fergal Martin, Swati Sinha, Leanne Haggerty, Chiara Bortoluzzi

**Affiliations:** 1 Biosciences, University of Exeter, Exeter, EX4 4SB, UK; 2 Department of Biology, Via U. Bassi, University of Padova, Padova, 58/B, Italy; 3 Leibniz Institute for the Analysis of Biodiversity Change, Museum Koenig Bonn, Bonn, 53113, Germany; 4 Natural History Museum, P.O. Box 1172, Blindern, University of Oslo, Oslo, 0318, Norway; 5 Centre for Ecological & Evolutionary Synthesis, University of Oslo, Oslo, Norway; 6 Department of Biological Sciences, University of New Brunswick Saint John, Saint John, Canada; 7 Tjärnö Marine Laboratory, University of Gothenburg, Gothenburg, Sweden; 8 Centre for Coastal Research, University of Agder, Kristiansand, Norway; 9 Tree of Life, Wellcome Sanger Institute, Hinxton, UK; 10 Wellcome Genome Campus, European Molecular Biology Laboratory, European Bioinformatics Institute, Hinxton, UK; 11 SIB Swiss Institute of Bioinformatics, Amphipôle, Quartier UNIL-Sorge, Lausanne, 1015, Switzerland

**Keywords:** Gambusia holbrooki, genome assembly, European Reference Genome Atlas, Biodiversity Genomics Europe, Earth Biogenome Project, Eastern mosquitofish, Poeciliidae, invasive alien species

## Abstract

The *Gambusia holbrooki* (eastern mosquitofish) reference genome will offer a crucial resource for understanding the evolution and adaptation of invasive freshwater fish species. The genome of *G. holbrooki* was assembled into two haplotypes through a phased assembly approach; however, only the primary haplotype was designated as the reference genome for annotation and downstream analyses. The entirety of the genome sequence was assembled into 24 contiguous chromosomal pseudomolecules and 1 mitochondrial genome. This chromosome-level assembly encompasses 0.67 Gb, composed of 421 contigs and 318 scaffolds, with contig and scaffold N50 values of 15.9 Mb and 29.6 Mb, respectively.

## Introduction

*Gambusia holbrooki*, a member of the *Poeciliidae* family, is a freshwater fish native to eastern North America ( Pyke, 2005). Along with its sister species *Gambusia affinis*, it has been introduced globally as a means of mosquito control, with often severe impacts on local biodiversity ( Fryxell *et al.*, 2022). In Europe, *Gambusia holbrooki* was introduced to Spain and Italy in 1921 and has since become established throughout the Southern part of the European continent ( Fryxell *et al.*, 2022). Mosquitofish ( *G. holbrooki* and *G. affinis*) can have devastating impacts on local biodiversity and has been listed among the top 100 invasive alien species ( Lowe *et al.*, 2000). Being omnivorous and tolerant to a variety of environmental conditions, mosquitofish quickly spread with negative consequences for local biodiversity. In addition to its well-documented invasion history, the mosquitofish is a longstanding model system for life history evolution research and was key in the development of life history theory ( Stearns, 1983). *Gambusia holbrooki* is listed as Least Concern (LC) in the IUCN Red List on Threatened Species (last assessed in 2012).

The generation of this reference resource was coordinated by the European Reference Genome Atlas (ERGA) initiative’s Biodiversity Genomics Europe (BGE) project, supporting ERGA’s aims of promoting transnational cooperation to promote advances in the application of genomics technologies to protect and restore biodiversity ( Mazzoni *et al.*, 2023).

## Materials & methods

ERGA's sequencing strategy includes Oxford Nanopore Technology (ONT) and/or Pacific Biosciences (PacBio) for long-read sequencing, along with Hi-C sequencing for chromosomal architecture, Illumina Paired-End (PE) for polishing (i.e. recommended for ONT-only assemblies), and RNA sequencing for transcriptomic profiling, to facilitate genome assembly and annotation.

### Sample and sampling information

On 24 July 2024, Clelia Gasparini and Francesco Santi sampled 8 male specimens of *Gambusia holbrooki*, which were identified based on morphology using the gonopodium structure ( Lloyd & Tomasov, 1985) at the University of Padua, Italy. Specimens were sampled in the Piovego canal (which connects the Bacchiglione and Brenta rivers), Padua, Italy. *Gambusia holbrooki* is the only mosquitofish species that has so far been described in Italy. Sampling was performed under permission number 382 (16 May 2024) issued by the Regione del Veneto, Italy. Specimens were caught with a dip net and immediately placed into a collection container. Specimens were then euthanized with an overdose of MS222 and subsequently put in dry ice. Until DNA extraction, samples were preserved at -80 °C.

### Vouchering information

Physical reference materials for the here sequenced specimen have been deposited in the Leibniz Institute for the Analysis of Biodiversity Change https://leibniz-lib.de/en/index.html under accession numbers ZFMK-TIS-97651 (male organism) and ZFMK-TIS-97652 (female organism).

Frozen somatic animal tissue is available from proxy individuals at the Biobank of the Leibniz Institute for the Analysis of Biodiversity Change https://leibniz-lib.de/en/index.html under the voucher IDs ZFMK-TIS-97677, ZFMK-TIS-97679, ZFMK-TIS-97680, ZFMK-TIS-97682 to ZFMK-TIS-97685, ZFMK-TIS-97688, ZFMK-TIS-97689, ZFMK-TIS-97692. Additionally, somatic cell cultures were established and frozen in liquid nitrogen under the ID ZFMK-TIS-98713.

### Genetic information

The estimated genome size, based on ancestral taxa, is 0.83 Gb. This is a diploid genome with a haploid number of 24 chromosomes (2n=48) and XX/XY as sex determination mechanism ( Kottler *et al.*, 2020). All information for this species was retrieved from Genomes on a Tree ( Challis *et al.,* 2023).

### DNA/RNA processing

Protocols for high molecular weight (HMW) DNA extraction developed at the Wellcome Sanger Institute (WSI) Tree of Life Core Laboratory are available on protocols.io ( Denton *et al*., 2023b; Howard *et al.*, 2025). The fGamHol4 sample was weighed and triaged ( Jay *et al*., 2023) to determine the appropriate extraction protocol. Tissue from the mid-body was homogenised by powermashing using a PowerMasher II tissue disruptor ( Denton *et al.,* 2023a). HMW DNA was extracted using the Automated MagAttract v2 protocol ( Oatley *et al.,* 2025a). DNA was sheared into an average fragment size of 12–20 kb following the Megaruptor®3 for LI PacBio protocol ( Bates *et al.,* 2023). Sheared DNA was purified by automated SPRI (solid-phase reversible immobilisation) ( Oatley *et al.*, 2025b). The concentration of the sheared and purified DNA was assessed using a Nanodrop spectrophotometer and Qubit Fluorometer using the Qubit dsDNA High Sensitivity Assay kit. Fragment size distribution was evaluated by running the sample on the FemtoPulse system.

### Library preparation and sequencing

Library preparation and sequencing were performed at the WSI Scientific Operations core. Libraries were prepared using the SMRTbell Prep Kit 3.0 (Pacific Biosciences, California, USA), according to the manufacturer’s instructions. The kit includes reagents for end repair/A-tailing, adapter ligation, post-ligation SMRTbell bead clean-up, and nuclease treatment. Size selection and clean-up were performed using diluted AMPure PB beads (Pacific Biosciences). DNA concentration was quantified using a Qubit Fluorometer v4.0 (ThermoFisher Scientific) and the Qubit 1X dsDNA HS assay kit. Final library fragment size was assessed with the Agilent Femto Pulse Automated Pulsed Field CE Instrument (Agilent Technologies) using the gDNA 55 kb BAC analysis kit.

The sample was sequenced on a Revio instrument (Pacific Biosciences). The prepared library was normalised to 2 nM, and 15 μL was used for making complexes. Primers were annealed and polymerases bound to generate circularised complexes, following the manufacturer’s instructions. Complexes were purified using 1.2X SMRTbell beads, then diluted to the Revio loading concentration (200–300 pM) and spiked with a Revio sequencing internal control. The sample was sequenced on a Revio 25M SMRT cell. The SMRT Link software (Pacific Biosciences), a web-based workflow manager, was used to configure and monitor the run and to carry out primary and secondary data analysis.

Biotinylated DNA constructs were fragmented using a Covaris E220 sonicator and size selected to 400–600 bp using SPRISelect beads. DNA was enriched with Arima-HiC v2 kit Enrichment beads. End repair, A-tailing, and adapter ligation were carried out with the NEBNext Ultra II DNA Library Prep Kit (New England Biolabs), following a modified protocol where library preparation occurs while DNA remains bound to the Enrichment beads. Library amplification was performed using KAPA HiFi HotStart mix and a custom Unique Dual Index (UDI) barcode set (Integrated DNA Technologies). Depending on sample concentration and biotinylation percentage determined at the crosslinking stage, libraries were amplified with 10–16 PCR cycles. Post-PCR clean-up was performed with SPRISelect beads. Libraries were quantified using the AccuClear Ultra High Sensitivity dsDNA Standards Assay Kit (Biotium) and a FLUOstar Omega plate reader (BMG Labtech). Prior to sequencing, libraries were normalised to 10 ng/μL. Normalised libraries were quantified again and equimolar and/or weighted 2.8 nM pools. Pool concentrations were checked using the Agilent 4200 TapeStation (Agilent) with High Sensitivity D500 reagents before sequencing. Sequencing was performed using paired-end 150 bp reads on the Illumina NovaSeq X. In total 56x HiFi and 147x HiC data were sequenced to generate the assembly.

### Genome assembly methods

The HiFi reads were assembled using Hifiasm ( Cheng *et al.*, 2021) in Hi-C phasing mode, where data were separated into two haplotypes. These haplotypes were then curated to generate a final assembly. The Hi-C reads were aligned to the contigs using bwa-mem2 ( Vasimuddin *et al.*, 2019), and contigs were scaffolded with YaHS ( Zhou *et al.*, 2023), using the --break option for handling potential misassemblies. The resulting scaffolded assemblies were evaluated using Gfastats ( Formenti *et al.*, 2022), BUSCO ( Manni *et al.*, 2021), and MERQURY.FK ( Rhie *et al.*, 2020).

The mitochondrial genome was assembled using oatk ( Zhou *et al.*, 2024) as a single circular contig of 18,667 bp and it is included in the released assembly (GCA_965178065.1). Manual curation was primarily conducted using PretextView ( Harry, 2022), with additional insights provided by JBrowse2 ( Diesh *et al.*, 2023) and HiGlass ( Kerpedjiev *et al.*, 2018). Scaffolds were visually inspected and corrected as described by ( Howe *et al.*, 2021). Any identified contamination, missed joins, and mis-joins were corrected, and duplicate sequences were tagged and removed. The curation process is documented at ( Denton *et al.*, 2023) (article in preparation). Summary analysis of the released assembly was performed using the ERGA-BGE Genome Report ASM Galaxy workflow ( doi.org/10.48546/workflowhub.workflow.1104.1).

### Genome annotation methods

A gene set was generated using the Ensembl Gene Annotation system ( Aken *et al.*, 2016), primarily by aligning publicly available short-read RNA-seq data from BioSample: SAMN14167175, SAMN14167173, SAMN11245730, SAMN11245729, SAMN11245733, SAMN11245732, SAMN14167176, SAMN14167159, SAMN14167158, SAMN11245734, SAMN11245731, SAMN14167178, SAMN14167177, SAMN14167174, SAMN14167160, SAMN14167157, SAMN14167156, and SAMN14167155 to the genome. Gaps in the annotation were filled via protein-to-genome alignments of a select set of vertebrate proteins from ( UniProt Consortium, 2019), which had experimental evidence at the protein or transcript level. At each locus, data were aggregated and consolidated, prioritising models derived from RNA-seq data, resulting in a final set of gene models and associated non-redundant transcript sets. To distinguish true isoforms from fragments, the likelihood of each open reading frame (ORF) was evaluated against known vertebrate proteins. Low-quality transcript models, such as those showing evidence of fragmented ORFs, were removed. In cases where RNA-seq data were fragmented or absent, homology data were prioritised, favouring longer transcripts with strong intron support from short-read data. The resulting gene models were classified into three categories: protein-coding, pseudogene, and long non-coding. Models with hits to known proteins and few structural abnormalities were classified as protein-coding. Models with hits to known proteins but displaying abnormalities, such as the absence of a start codon, non-canonical splicing, unusually small intron structures (<75 bp), or excessive repeat coverage, were reclassified as pseudogenes. Single-exon models with a corresponding multi-exon copy elsewhere in the genome were classified as processed (retrotransposed) pseudogenes. Models that did not fit any of the previously described categories did not overlap protein-coding genes and were constructed from transcriptomic data were considered potential lncRNAs. Potential lncRNAs were further filtered to remove single-exon loci due to their unreliability. Putative miRNAs were predicted by performing a BLAST search of miRBase ( Kozomara *et al.*, 2019) against the genome, followed by RNAfold analysis ( Gruber *et al.*, 2008). Other small non-coding loci were identified by scanning the genome with Rfam ( Kalvari *et al.*, 2018) and passing the results through Infernal ( Nawrocki & Eddy, 2013). Summary analysis of the released annotation was carried out using the ERGA-BGE Genome Report ANNOT Galaxy workflow ( 10.48546/workflowhub.workflow.1096.1).

## Results

### Genome assembly

**Figure 1.**
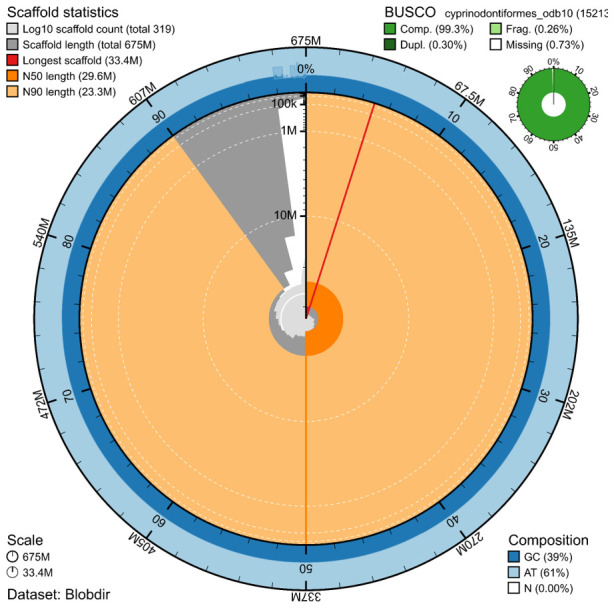
Snail plot summary of assembly statistics. The main plot is divided into 1,000 size-ordered bins around the circumference, with each bin representing 0.1% of the 674,622,098 bp assembly including the mitochondrial genome. The distribution of sequence lengths is shown in dark grey, with the plot radius scaled to the longest sequence present in the assembly (33.4 Mp, shown in red). Orange and pale-orange arcs show the scaffold N50 and N90 sequence lengths (29,628,722 and 23,341,799 bp), respectively. The pale grey spiral shows the cumulative sequence count on a log-scale, with white scale lines showing successive orders of magnitude. The blue and pale-blue area around the outside of the plot shows the distribution of GC, AT, and N percentages in the same bins as the inner plot. A summary of complete, fragmented, duplicated, and missing BUSCO genes found in the assembled genome from the Cyprinodontiformes database (odb10) is shown in the top right.

**Figure 2.**
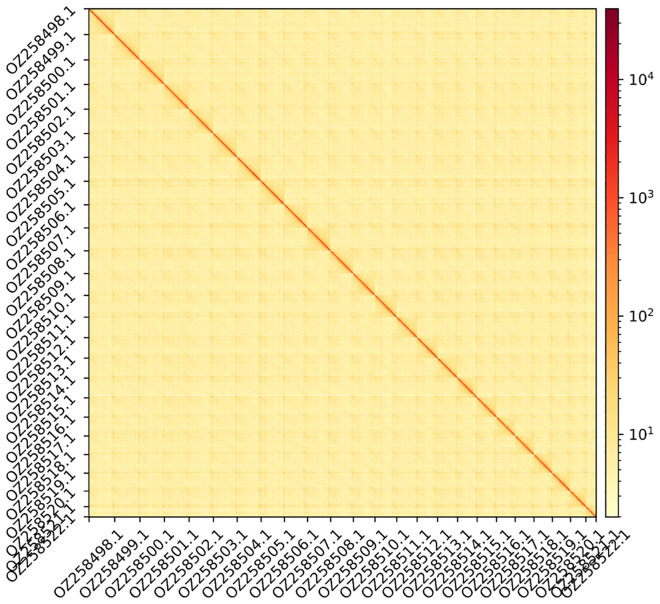
Hi-C contact map showing spatial interactions between regions of the genome. The diagonal corresponds to intra-chromosomal contacts, depicting chromosome boundaries. The frequency of contacts is shown on a logarithmic heatmap scale. Hi-C matrix bins were merged into a 200 kb bin size for plotting.

The genome assembly has a total length of 674,622,098 bp in 318 scaffolds including the mitogenome ( Figure 1 & Figure 2), with a GC content of 39%. The assembly has a contig N50 of 15,918,108 bp and L50 of 16 and a scaffold N50 of 29,628,722 bp and L50 of 11. The assembly has a total of 103 gaps, totalling 11 kb in cumulative size. The single-copy gene content analysis using the Cyprinodontiformes database (odb10) with BUSCO ( Manni *et al.*, 2021) resulted in 99.3% completeness (99.0% single and 0.3% duplicated). 93.8% of reads k-mers were present in the assembly and the assembly has a base accuracy Quality Value (QV) of 59.8 as calculated by Merqury ( Rhie *et al.*, 2020).

### Genome annotation

The genome annotation consists of 21,050 protein-coding genes with associated 56,794 transcripts, in addition to 3,687 non-coding genes ( Table 1). Using the longest isoform per transcript, the single-copy gene content analysis using the Cyprinodontiformes database (odb10) database with BUSCO resulted in 88.5% completeness. Using the OMAmer Metazoa-v2.0.0.h5 database for OMArk ( Nevers *et al.*, 2025) resulted in 94.0% completeness and 98.5% consistency ( Table 2).

**Table 1.** Statistics from assembled gene models.

	No. genes	No. transcripts	Mean gene length (bp)	No. single- exon genes	Mean exons per transcript
**mRNA**	21,050	56,794	18,920	517	14.3
**pseudogene**	228	228	22,143	1	24.5
**snoRNA**	158	158	114	158	1.0
**lncRNA**	2,085	2,520	4,158	1,283	2.6
**miRNA**	25	25	69	25	1.0
**snRNA**	118	118	153	118	1.0
**rRNA**	1,066	1,066	612	1,066	1.0
**scRNA**	7	7	223	7	1.0
**Other ncRNA**	13	13	98 - 275	13	1.0 - 1.0

**Table 2.** Annotation completeness and consistency scores calculated by BUSCO run in protein mode (cyprinodontiformes_odb10) and OMArk (Metazoa-v2.0.0.h5).

	Complete	Singular	Duplicated	Fragmented	Missing
**BUSCO**	13,470 (88.5%)	13,391 (88.0%)	79 (0.5%)	197 (1.3%)	1,546 (10.2%)
**OMArk**	17,336 (94.0%)	17,053 (92.5%)	283 (1.5%)	-	1,105 (5.6%)
	Consistent	Inconsistent	Contaminants	Unknown	
**OMArk**	20,764 (98.5%)	142 (0.7%)	0.0 (0.0%)	166 (0.8%)	

## Data Availability

*Gambusia holbrooki* and the related genomic study were assigned to Tree of Life ID (ToLID) 'fGamHol4' and all sample, sequence, and assembly information are available under the umbrella BioProject PRJEB84145. The sample information is available at the following BioSample accessions: SAMEA115931355 and SAMEA115931357. The genome assembly is accessible from ENA under accession number GCA_965282415.1 and the annotated genome is available through the Ensembl website ( https://projects.ensembl.org/erga-bge/). Sequencing data produced as part of this project are available from ENA at the following accessions: ERX13553727 and ERX14241339. Documentation related to the genome assembly and curation can be found in the ERGA Assembly Report (EAR) document available at https://github.com/ERGA-consortium/EARs/tree/main/Assembly_Reports/Gambusia_holbrooki/fGamHol4. Further details and data about the project are hosted on the ERGA portal at https://portal.erga-biodiversity.eu/data_portal/37273.
