# Supplementary material for: ERGA-BGE reference genome of Gambusia holbrooki, a globally invasive freshwater fish
Source: Open Res Eur. 2026 Jun 4;6:48. Originally published 2026 Feb 13. [Version 2] doi: 10.12688/openreseurope.22693.2 (PMC13291747; doi:10.12688/openreseurope.22693.2)
Supplement: Peer review history

# Reviewer report: Reviewer response for version 2

**DOI:** 10.21956/openreseurope.26126.r75454

**Published:** 2026-06-23

**Authors:** Roger Huerlimann

**Affiliations:** 1 Okinawa Institute of Science and Technology, Okinawa, Japan

The authors have addressed all of my concerns, and I approve the latest version for acceptance.

Are sufficient details of methods and materials provided to allow replication by others?

Yes

Is the rationale for creating the dataset(s) clearly described?

Yes

Are the datasets clearly presented in a useable and accessible format?

Yes

Are the protocols appropriate and is the work technically sound?

Yes

Reviewer Expertise:

NA

I confirm that I have read this submission and believe that I have an appropriate level of expertise to confirm that it is of an acceptable scientific standard.

# Reviewer report: Reviewer response for version 2

**DOI:** 10.21956/openreseurope.26126.r75453

**Published:** 2026-06-12

**Authors:** Alejandro S. Mechaly

**Affiliations:** 1 Instituto de Investigaciones en Biodiversidad y Biotecnología (INBIOTEC-CONICET), Mar del Plata, Argentina

The manuscript is now suitable for indexing. The authors have adequately addressed my previous concerns and have implemented the suggested revisions in a satisfactory manner. I have no further major comments and recommend indexing of the manuscript in its current form.

Are sufficient details of methods and materials provided to allow replication by others?

Yes

Is the rationale for creating the dataset(s) clearly described?

Yes

Are the datasets clearly presented in a useable and accessible format?

Yes

Are the protocols appropriate and is the work technically sound?

Yes

Reviewer Expertise:

NA

I confirm that I have read this submission and believe that I have an appropriate level of expertise to confirm that it is of an acceptable scientific standard.

# Reviewer report: Reviewer response for version 1

**DOI:** 10.21956/openreseurope.24541.r69741

**Published:** 2026-03-19

**Authors:** Roger Huerlimann

**Affiliations:** 1 Okinawa Institute of Science and Technology, Okinawa, Japan

This data note describes a chromosome-level reference genome assembly for *Gambusia holbrooki* (eastern mosquitofish), a globally invasive freshwater fish native to eastern North America, produced under the ERGA-BGE framework.

 The male specimen was sampled from a canal in Padua, Italy, and sequenced using PacBio HiFi and Hi-C technologies. The genome was assembled using Hifiasm in Hi-C phasing mode, producing a phased, two-haplotype assembly, with the primary haplotype designated as the reference. The final assembly spans about 675 Mb organized into 24 chromosomal pseudomolecules plus a mitochondrial genome, with a contig N50 of 15.9 Mb and scaffold N50 of 29.6 Mb. BUSCO completeness at the assembly level was 99.3%, indicating a high-quality result.

 Genome annotation identified 21,050 protein-coding genes and 3,687 non-coding genes, though annotation-level BUSCO completeness was lower at 88.5%. I agree with the other reviewer that this should be briefly addressed by the authors. 

 While this gap may partly reflect the conservative filtering steps applied during annotation, such as removal of fragmented ORFs and low-quality transcript models, the authors should briefly discuss the primary drivers of this discrepancy. Specifically, it would be useful to know whether the gap reflects limitations in the available RNA-seq data, the phylogenetic distance of the reference protein set used for gap-filling, the stringency of the Ensembl pipeline's filtering criteria, or a combination of these factors. Clarifying whether the lower annotation BUSCO score represents genuine missing gene content or is an expected outcome of conservative quality filtering would improve transparency and help readers interpret the annotation completeness metrics appropriately.

 The protocols used are appropriate for this kind of work, and they are well described in the material and methods section. The data availability statement is also providing all the necessary information.

 One minor comment about the first paragraph in the M&M section. I don't think it's necessary to mention ERGA's general sequencing strategy. Mentioning ONT and Illumina short read polishing even though these have not been used in this assembly might confuse readers.

Are sufficient details of methods and materials provided to allow replication by others?

Yes

Is the rationale for creating the dataset(s) clearly described?

Yes

Are the datasets clearly presented in a useable and accessible format?

Yes

Are the protocols appropriate and is the work technically sound?

Yes

Reviewer Expertise:

My research spans marine genomics, transcriptomics, and eDNA metabarcoding in non-model fish and invertebrate species. I have hands-on experience with genome assembly and annotation, including chromosome-level assemblies of multiple fish species and the black tiger prawn, using a range of sequencing technologies and assembly approaches. I am familiar with standard quality assessment metrics including BUSCO, and with RNA-seq-based annotation workflows.

I confirm that I have read this submission and believe that I have an appropriate level of expertise to confirm that it is of an acceptable scientific standard, however I have significant reservations, as outlined above.

# Response

**Published:** 2026-05-26

**Authors:** Chiara Bortoluzzi

**Affiliations:** Swiss Institute of Bioinformatics, Lausanne, Vaud, Switzerland

We thank the reviewer for their comments. We addressed as follow:

1. We thank the reviewer for their comment. As reported for reviewer #1, the annotation was generated using the automated Ensembl annotation pipeline, which applies relatively stringent filtering criteria during gene model construction. In particular, transcript-supported models are prioritised when they are additionally supported by protein homology evidence, including similarity searches against curated UniProt protein datasets. Such conservative filtering can reduce false-positive annotations but may also exclude partial, lowly expressed, lineage-specific, or weakly supported gene models, thereby lowering annotation BUSCO completeness relative to the assembly BUSCO score. In addition, the annotation relied primarily on short-read RNA-seq data, which may not fully capture transcript diversity, splice isoforms, or genes expressed only in specific tissues or developmental stages. The incorporation of broader transcriptomic resources, particularly long-read RNA sequencing and more diverse tissue sampling, could improve gene model completeness in future annotation releases. Alternative or complementary annotation strategies with less conservative filtering may also recover additional BUSCO genes
2. We removed the section on ONT data as suggested.

# Reviewer report: Reviewer response for version 1

**DOI:** 10.21956/openreseurope.24541.r69744

**Published:** 2026-02-28

**Authors:** Alejandro S. Mechaly

**Affiliations:** 1 Instituto de Investigaciones en Biodiversidad y Biotecnología (INBIOTEC-CONICET), Mar del Plata, Argentina

This Data Note presents a chromosome-level reference genome assembly for *Gambusia holbrooki* generated within the ERGA-BGE framework. The manuscript is clearly written, technically rigorous, and fully consistent with the scope of a genome report. The final assembly spans 674.6 Mb organized into 24 chromosomal pseudomolecules plus the mitochondrial genome, with a contig N50 of 15.9 Mb and a scaffold N50 of 29.6 Mb. The reported BUSCO completeness of 99.3% at the assembly level and a QV of 59.8 indicate high contiguity and excellent base-level accuracy. The Hi-C contact map further supports appropriate chromosomal organization and structural reliability. Overall, the assembly meets current standards for a high-quality reference genome.

 The annotation comprises 21,050 protein-coding genes and 3,687 non-coding genes. While the assembly-level BUSCO completeness is very high (99.3%), the protein-based BUSCO score for the annotation (88.5%) is noticeably lower. Although acceptable for an automated Ensembl-based pipeline, this difference is relatively pronounced for a long-read, chromosome-scale assembly of moderate genome size. The authors are encouraged to briefly discuss the reasons for this discrepancy and clarify whether it reflects annotation stringency, limited transcriptomic diversity, conservative filtering criteria, or other methodological factors. Indicating whether additional transcriptomic resources or alternative annotation strategies could improve gene completeness would strengthen the transparency of the manuscript.

 The methodological description is detailed and reproducible, and sampling, vouchering, and data accessibility are well documented. The study clearly adheres to ERGA standards and provides a valuable genomic resource for a globally invasive and evolutionarily important species.

 From a biological and comparative perspective, the manuscript would benefit from slightly stronger contextualization. First, incorporating reference to confirmed records of *Gambusia holbrooki* in South America, particularly Argentina—where taxonomic confusion with *G. affinis* has been documented (Cabrera et al., 2017, *Journal of Fish Biology* 91:704–710)—would strengthen the biogeographic and invasion framework of the Introduction and provide a more complete overview of the species’ global introduction history.

 Second, the authors may wish to position this assembly within the rapidly expanding genomic resources available for Poeciliidae. Recent chromosome-level genome assemblies for multiple

*Poecilia* and *Gambusia* lineages (Ryan et al., 2025, *Genome Biology and Evolution* 17:e vaf111) highlight the growing comparative framework within the family. A concise comparison of assembly metrics and a brief discussion of how the present genome complements existing resources would help contextualize its contribution.

 Overall, this is a technically robust and well-documented reference genome assembly that will serve as a useful resource for ecological, evolutionary, and comparative genomics. I recommend acceptance after minor revision.

Are sufficient details of methods and materials provided to allow replication by others?

Yes

Is the rationale for creating the dataset(s) clearly described?

Yes

Are the datasets clearly presented in a useable and accessible format?

Yes

Are the protocols appropriate and is the work technically sound?

Yes

Reviewer Expertise:

Specialized in fish biology, neuroendocrinology, and comparative genomics, this research focuses on the molecular and physiological mechanisms underlying reproduction and adaptation in aquatic vertebrates. Endocrine physiology is integrated with genome assembly and transcriptomic analyses to investigate sex determination, population differentiation, and responses to environmental stressors, contributing to both fundamental biological knowledge and sustainable aquaculture development.

I confirm that I have read this submission and believe that I have an appropriate level of expertise to confirm that it is of an acceptable scientific standard, however I have significant reservations, as outlined above.

# Response

**Published:** 2026-05-26

**Authors:** Chiara Bortoluzzi

**Affiliations:** Swiss Institute of Bioinformatics, Lausanne, Vaud, Switzerland

We thank the reviewer for their comments. We addressed them as follow: 

1. We thank the reviewer for this important observation. The discrepancy between the very high assembly-level BUSCO completeness (99.3%) and the lower protein-based BUSCO score for the annotation likely reflects characteristics of the automated annotation workflow. The annotation was generated using the automated Ensembl annotation pipeline, which applies relatively stringent filtering criteria during gene model construction. In particular, transcript-supported models are prioritised when they are additionally supported by protein homology evidence, including similarity searches against curated UniProt protein datasets. Such conservative filtering can reduce false-positive annotations but may also exclude partial, lowly expressed, lineage-specific, or weakly supported gene models, thereby lowering annotation BUSCO completeness relative to the assembly BUSCO score. In addition, the annotation relied primarily on short-read RNA-seq data, which may not fully capture transcript diversity, splice isoforms, or genes expressed only in specific tissues or developmental stages. The incorporation of broader transcriptomic resources, particularly long-read RNA sequencing and more diverse tissue sampling, could improve gene model completeness in future annotation releases. Alternative or complementary annotation strategies with less conservative filtering may also recover additional BUSCO genes
2. We have now added the introduction of G. holbrooki in Argentina and Australia along with Europe. As well as the taxonomic confusion with G. affinis. 
3. We added a reference to the GBE paper in the introduction as suggested.
